# Components of day-to-day variability of cerebral perfusion measurements – Analysis of phase contrast mapping magnetic resonance imaging measurements in healthy volunteers

**DOI:** 10.1371/journal.pone.0197807

**Published:** 2018-06-07

**Authors:** Abd R. A. Ismaili, Mark B. Vestergaard, Adam E. Hansen, Henrik B. W. Larsson, Helle H. Johannesen, Ian Law, Otto M. Henriksen

**Affiliations:** 1 Department of Clinical Physiology, Nuclear Medicine & PET, Rigshospitalet Blegdamsvej, University of Copenhagen, Copenhagen, Denmark; 2 Institute for Clinical Medicine, The Faculty of Health and Medical Sciences, University of Copenhagen, Copenhagen, Denmark; Boston University, UNITED STATES

## Abstract

**Purpose:**

The aim of the study was to investigate the components of day-to-day variability of repeated phase contrast mapping (PCM) magnetic resonance imaging measurements of global cerebral blood flow (gCBF).

**Materials and methods:**

Two dataset were analyzed. In Dataset 1 duplicated PCM measurements of total brain flow were performed in 11 healthy young volunteers on two separate days applying a strictly standardized setup. For comparison PCM measurements obtained from a previously published study (Dataset 2) were analyzed in order to assess long-term variability in an aged population in a less strictly controlled setup. Global CBF was calculated by normalizing total brain flow to brain volume. On each day measurements of hemoglobin, caffeine and glucose were obtained. Linear mixed models were applied to estimate coefficients of variation (CV) of total (CV_t_), between-subject (CV_b_), within-subject day-to-day (CV_w_), and intra-session residual variability (CV_r_).

**Results:**

In Dataset 1 CV_t_, CV_b_, CV_w_ and CV_r_ were estimated to be 11%, 9.4%, 4% and 4.2%, respectively, and to 8.8%, 7.2%, 2.7% and 4.3%, respectively, when adjusting for hemoglobin and plasma caffeine. In Dataset 2 CV_t_, CV_b_ and CV_w_ were estimated to be 25.4%, 19.2%, and 15.0%, respectively, and decreased to 16.6%, 8.2% and 12.5%, respectively, when adjusting for the same covariates.

**Discussion:**

Our results suggest that short-term day-to-day variability of gCBF is relatively low compared to between-subject variability when studied in standardized conditions, whereas long-term variability in an aged population appears to be much larger when studied in less a standardized setup. The results further showed that from 20% to 35% of the total variability in gCBF can be attributed to the effects of hemoglobin and caffeine.

## Introduction

Quantitative measurements of global cerebral blood flow (gCBF) are of general interest both for basic physiological investigations and for our understanding of brain aging and the pathophysiology of brain diseases [1] Many brain diseases are associated with altered gCBF, and several studies have reported that decreased total brain flow or gCBF may be associated with severity of vascular lesions and brain atrophy [2], impaired cognitive function [3] and may even predict overall future mortality in old age [4]. However, in order to fully interpret these results, more detailed knowledge of the normal variability between and within individuals is required.

Previous studies investigating variability of gCBF measurements have mainly focused on separating between-subject variability from residual intra-session variability considered to reflect method imprecision [5–7]. These studies have confirmed large and similar between-subject variability, whereas intra-session variability varies substantially depending on the method applied. However, when obtaining measurements at two different time-points, the within-subject variability consists of both the true inter-session (day-to-day) variability and the intra-session variability (imprecision). Little is known about the normal temporal variability of gCBF and the factors influencing this variability. Like most other physiological parameters gCBF is expected to exhibit temporal variations, probably with both high-frequency (seconds to minutes) variations reflecting variations in blood pressure and respiration pattern [8], low frequency variations related to diurnal variations [9] and sleep [10], very low frequency variations related to menstrual cycle [11], and also age related changes [12]. Accordingly, intra-subject variability is expected to increase over time and may also depend on both the population studied and the experimental setup.

In cross-sectional studies it is of key interest to know how representative a single gCBF measurements is for a particular participant, i.e. how much would gCBF vary if the measurement was obtained on a different day? Establishing normal variability over time is therefore essential for interpreting the association of gCBF with various factors or of temporal changes in gCBF, and such knowledge is also useful for planning studies involving measurement of gCBF.

It is generally assumed that cerebral metabolic rate of oxygen should be relatively constant during resting awake conditions, and consequently the delivery of oxygen to the brain should exhibit low temporal variability [13]. However, a number of factors may influence the relationship between cerebral blood supply and metabolism. The hemoglobin concentration has been shown to be a major determinant of gCBF [14], but is expected to show minimal temporal variability and thus mainly influence between-subject variability. In contrast, caffeine, which is widely consumed in the public, may decrease gCBF by up to 30% after dietary intake in normal quantities [15, 16], and may thus be an important cause of day-to-day variation in gCBF [17].

The aims of this study were to estimate the components of the day-to-day variability of repeated measurements of gCBF and to assess the influence of hemoglobin and plasma caffeine levels on these components. Further, we wished to compare variability of short-term repeated measurements obtained in strictly controlled conditions with long-term variability in an aged population under less well-controlled conditions.

## Materials and methods

Day-to-day variability was investigated in two separate datasets. In Dataset 1 short-term variability was investigated in healthy young male volunteers examined in standardized conditions with duplicate PCM measurements performed on each of two study days < 2 weeks apart. In Dataset 2 long term-variability was assessed from single PCM measurements in elderly, healthy volunteers performed on two study days > 3 months apart. Data are provided as supporting information (S1 Data).

Both studies were approved by the Scientific Health Ethics Committee of the Capital region of Denmark (Ref. H-D-2008-002 and H-B-2008-075) following the standards of The National Committee on Health Research Ethics, and were conducted in accordance with the Helsinki Declaration. All volunteers gave informed written consent.

### Dataset 1

#### Subjects

A total of 12 healthy young male volunteers were included in the study (mean age 24 [range 21 to 28] years). One participant was subsequently excluded due to a detected structural abnormality. Eligible subjects had a caffeine intake of 1–5 cups of tea or coffee daily. Exclusion criteria comprised prior incidents of severe head trauma, a history of neurological, psychiatric, or endocrine disorders, or contraindications to MRI.

#### Study design

Duplicate measurements of total brain flow were performed on two separate days (1–16 days apart) using an identical imaging protocol. Venous blood samples were collected on each study day and analyzed for hemoglobin, glucose and caffeine. All scans were performed during the daytime by the same researcher (A.I).

#### MRI experiment

MRI scans were performed on a 3T hybrid PET/MR system (Biograph mMR, Siemens Healthcare, Erlangen, Germany) using a 16 channel receive head-neck coil. On each day MRI measurements were conducted within a single session of approximately 45 minutes. Participants were instructed to abstain from coffee or other methylxanthine containing beverages or food items six hours prior the experiment, and not to consume any food two hours prior to the scan.

**Fig 1:**
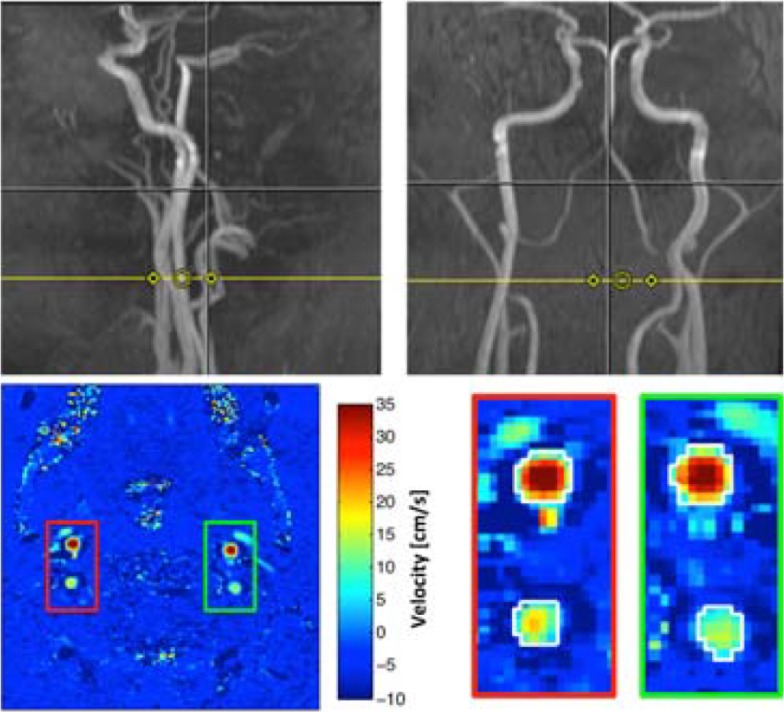
Planning and analysis of phase contrast mapping measurements in Dataset 1. Top panel shows planning of the PCM measurement slice (yellow line) on sagittal (left) and coronal (right) maximum intensity projections of cerebral arteries. Lower panels shows color coded velocity maps. The right (red) and left (blue) cerebral arteries are clearly visible on the mean velocity image. Lower right panel shows manually drawn regions of interest corresponding to each artery.

Initially a 3D inflow angiogram (64 slices per slab, voxel size 1.4×1.0×1.66 mm, TE = 6.04 ms, TR = 39.90 ms, flip angle 15°) of the cerebral vessels was obtained. Based on coronal and sagittal maximal intensity projections the PCM measurement slice was placed as perpendicular as possible to the vertebral and internal carotid arteries at the level of the third vertebral artery segment (Fig 1). Measurements were obtained with the following parameters (1 slice, slice thickness = 5.0 mm, voxel size 0.8×0.8×5.0 mm, TE = 3.38 ms, TR = 37.40 ms, flip angle 25°, 6 measurement, encoding velocity = 150 cm/s). The sequence was pulse triggered (retrospective gaiting 20 frames/cycle).

On one of the study days a structural scan for tissue segmentation and calculation of brain volume was acquired using a 3D T1 weighted gradient echo sequence (TR = 1900 ms, TE = 2.44 ms, flip angle 9°, voxel size: 1.0×1.0×1.0 mm^3^).

#### Image and data processing

PCM data were processed using in-house software based on Matlab (Mathwoks, Natick, MA). Regions of interest were manually drawn around the internal carotid and vertebral arteries on the mean velocity image (Fig 1). Flow in each vessel was calculated by multiplying mean velocity with vessel area and integrating over time. All processing was performed by the same investigator (A.I.).

For calculation of brain volume, the 3D T1 weighted scan was converted into NIfTI format and analyzed using the FSL tools BET and FAST (FMRIB Software Library, Oxford University, Oxford, UK). Total brain volume was calculated as the sum of the white and grey tissue volumes including also cerebellum and brain stem. Mean gCBF was calculated as the sum of flows (ml/min) in the four arteries (total blood flow) divided by the brain volume, and reported in ml/100ml/min. The same segmented scan and brain volume was used for calculation of all gCBF values in each participant.

### Dataset 2

Repeated PCM measurements obtained as a part of a previously published study were analyzed for comparison. Single PCM measurements were performed on two study days > 3 months apart (range 140 to 252 days) in 13 aged healthy volunteers (6 men / 7 women, age 52–68 years) [18]. Repeated measurements have not been published previously. Study populations characteristics and experimental design including MRI sequence parameters and post-processing have been described in details previously [18]. Estimates of intrasession variability (CV_r_) was obtained from a previous study of young healthy volunteers [7]. Both studies were performed on a 3T Philips Achiva MR system using very similar imaging protocols (details are provided in S1 Table). Triggered PCM measurements of the basilar and internal carotid arteries were analyzed using in-house software based on Matlab. Brain volume was obtained from a 3D T1 scan as described above.

Participants were allowed to consume coffee and tea as usual, and blood samples for determination of plasma caffeine, blood hemoglobin and plasma glucose were obtained and analyzed as described above.

### Statistical analysis

All statistical analysis was performed using SAS version 9.4 (SAS Institute Inc., Cary, NC). Repeatability of repeated measurements was assessed by a Bland-Altman analysis, and a paired t-test was applied for significance testing of bias. In Dataset 1 we investigated both intra-session repeatability on study day 1 (measurement 1- measurement 2) and inter-session (or day-to-day) repeatability (measurement 1 day 1—measurement 1 day 2). For simplicity and for comparability with Dataset 2, only repeatability of the first measurement on day 1 is reported in Bland-Altman analysis, whereas all measurements were include in the mixed linear model below.

Total variance ($\sigma_{t}^{2}$) is the sum of between-subject ($\sigma_{b}^{2}$), within-subject ($\sigma_{w}^{2}$) and residual ($\sigma_{r}^{2}$) variance:

$$\sigma_{t}^{2}=\sigma_{b}^{2}+\sigma_{w}^{2}+\sigma_{r}^{2}$$

These variance components can be estimated along with regression coefficients of covariates using by applying a 3-level mixed linear model with subject (level 3) and day (within-subject, level 2) as random effects (and replicate measurement as residual, level 1), and measurement number and study day as fixed effects:

$${gCBF}_{i,j,k}={gCBF}_{pop}+measurement+day+\delta_{i}+\zeta_{i,j}+\varepsilon_{i,j,k},$$

where g*CBF*_*pop*_ represents the population mean and *δ*_*i*_, *ζ*_*j*,*i*_ and *ε*_*i*,*j*,*k*_ denote mean off-set of the *i*’th subject from population mean, offset from subject mean on the *j*’th day, and off-set of the *k*’th measurement from the subject day mean, respectively. Each of these random components can be described by a normal distribution with mean value of zero and a standard deviation of σ_b_, σ_w_, σ_r._, respectively. The corresponding coefficients of variation (CV_t_, CV_b_, CV_w_ and CV_r_) can be calculated as the standard deviation (σ) of each component divided by the mean value of all measurements. To assess the effects of co-variates on variability, hemoglobin and caffeine were successively added to the model as fixed effects. No effect of glucose on gCBF was observed, and glucose was not included in the final models.

Dataset 2 was analyzed as above, except that a 2-level linear mixed model was applied with study day as fixed effect. An additional model including also caffeine and hemoglobin as fixed effects was also investigated. A similar model was applied to data from ref. [7] (including only data from the 14 participants with duplicate measurements) to estimate same-day $\sigma_{r}^{2}$ and $\sigma_{w}^{2}$. In Dataset 2 residual variance equals the sum of $\sigma_{r}^{2}$ and $\sigma_{w}^{2}$, i.e. the total within-subjects variance ($\sigma_{tw}^{2}$) Under the assumption that method precision (intra-session CV_r_) is of similar relative size in Dataset 2 as in ref. [7], we can obtain an estimate of $\sigma_{r}^{2}$ in Dataset 2 (by multiplying population mean in Dataset 2 with CV_r_ determined from data in ref. [7]) which in turn can be subtracted from σ_tw_^2^ in Dataset 2 in order to obtain an estimate of $\sigma_{w}^{2}$ taking methods precision into account.

## Results

### Dataset 1

All volunteers completed successfully both study days. Data from one participant on one study day were excluded due to poor quality of PCM measurements. Characteristics of participants and MRI mean results are presented in Table 1.

**Table 1:** Mean study population values.

	Dataset 1		Dataset 2	
	Mean ± SD	Range	Mean ± SD	Range
**Hemoglobin (mmol/l)**	9.3 ± 0.6	7.8–9.9	8.7 ± 0.6	7.6–9.9
**Plasma caffeine (μmol/l)**	0.70 (0.14–1.26)†	0.03–9.6	25.3 (8.3–32.0)†	0.01–108
**Glucose (mmol/l)**	4.8 ± 0.5	3.8–6.3	5.6 ± 0.8	4.0–7.6
**Height (cm)**	183.3 ± 6.1	174–196	169.6 ± 10.0	156–186
**Weight (kg)**	82.0 ± 12.0	65–98	77.2 ± 16.0	57–116
**Body mass index (kg/m**^**2**^**)**	24.3 ± 2.8	20.3–28.7	26.5 ± 3.5	22.3–34.3
**Brain volume (ml)**	1157.1 ± 71.8	1033–1270	1073.0 ± 103.8	888–1242
**Total blood flow (ml/min)**	733.5 ± 88.3	566.5–915.1	539.58 ± 145.9	305.6–791.9
**Cerebral blood flow (ml/100ml/min)**	63.4 ± 6.9	50.5–76.6	50.5 ± 13.6	25.4–74.4

†Median (interquartile range).

**Fig 2:**
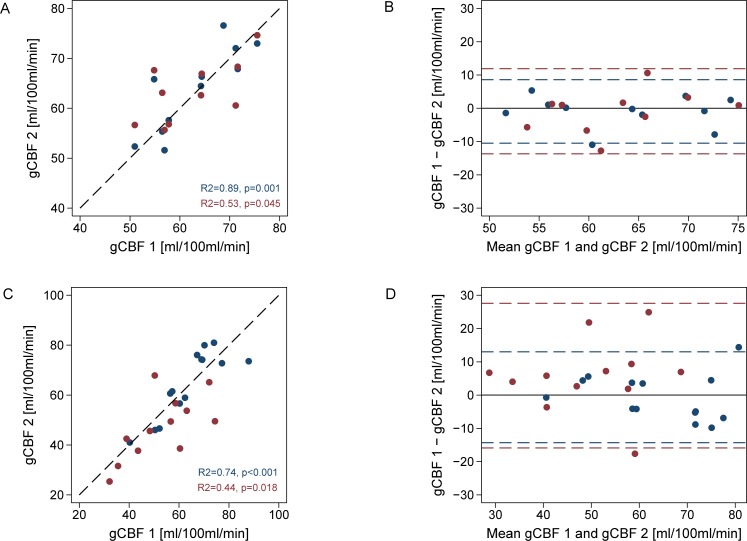
Repeatability of cerebral blood flow measurements. Scatter plots (left) and Bland-Altman plots (right) of repeated measurements from Dataset 1 (top panels) and Dataset 2 along with same-day measurements in young volunteers from ref. [7] (lower panels). Both measurements obtained on the same day (blue circles) and on different days (red circles) are shown. The dashed line in the scatter plots represents the line of identity and the dashed lines in the Bland-Altman plots represents the intra-session (blue) and inter-session (red) upper and lower limits of repeatability.

The Bland-Altman analysis showed slightly poorer day-to-day repeatability compared to within-day repeatability (Fig 2A and 2B) No significant bias between repeated measurements was observed.

**Fig 3:**
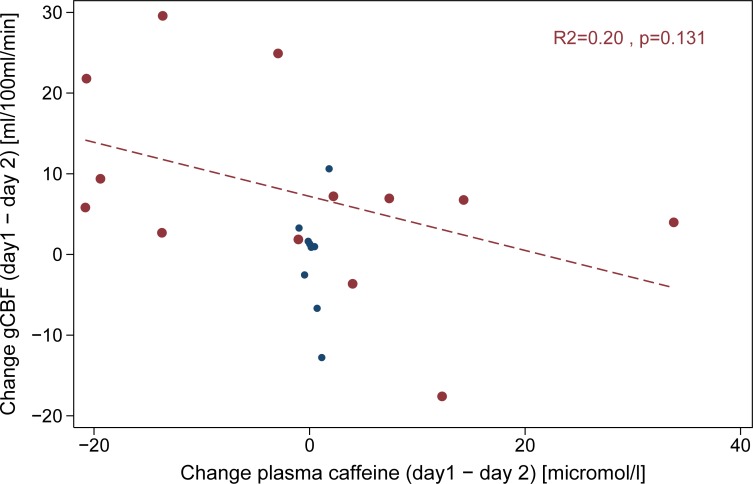
Influence of caffeine on day-to-day gCBF changes. Scatter plot showing change in gCBF and in plasma caffeine from day 1 to day 2 in standardized caffeine abstained state (Dataset 1, blue circles) and in spontaneous caffeinated state (Dataset 2, red circles and regression line). From Dataset 1 only the first measurement from each day is included.

In the final linear mixed model gCBF was inversely correlated with hemoglobin (-7.08 [95%CI: -11.35 to -2.82] ml/100ml/min per mmol/l, p = 0.002), and tended also to be inversely associated with caffeine (-0.98 [95%CI: -2.19 to 0.24] ml/100ml/min per **μ**mol/l, p = 0.107). Excluding a single outlying and influential measurement (with very high caffeine levels and low gCBF), the effect of caffeine on gCBF was further attenuated. Also, as the changes in plasma caffeine between days were all close to zero, no association of change in plasma caffeine with change in gCBF could be demonstrated (Fig 3).

The effects of adjusting for hemoglobin and caffeine on the total and individual components of variability are presented in Table 2. Generally, CV_b_ was found to be much larger than CV_w_ which in turn was slightly lower than CV_r_.

**Table 2:** Variance components of day-to-day variability of gCBF measurements.

	gCBF				Variability			
	Mean	σ_b_	σ_w_	σ_r_	CV_b_	CV_r_	CV_w_	CV_t_
	(ml/100ml/min)							
**Dataset 1**								
No covariates	64.3	5.9	2.5	2.6	9.4%	4.2%	4.0%	11.0%
Hgb	64.3	4.8	1.9	2.6	7.7%	4.2%	3.0%	9.2%
Hgb+caffeine	64.3	4.5	1.7	2.7	7.2%	4.3%	2.7%	8.8%
**Dataset 2**								
No covariates	50.5	9.69	-	8.37	19.2%	-	15.0%†	25.4%
Hgb+caffeine	50.5	4.14	-	7.27	8.2%	-	12.5%†	16.6%
**Data from ref. [7]**								
No covariates	64.9	10.98	-	4,63	16.9%	7.1%	-	18.4%

†Estimated using CV_r_ from ref. [7], see statistics section.

Abbreviations: gCBF = global cerebral blood flow, σ = standard deviation, CV = coefficient of variation, Hgb = hemoglobin. Subscripts *b*, *r* and *w* refer to between-subject variability, residual variability and within-subject variability, respectively.

CV_t_ decreased by 20% when including all covariates in the model. The reductions in total variability were predominantly due to corresponding reductions in CV_b_, which decreased by 23%. The inclusion of caffeine did only result in a small decrease in CV_w_. Otherwise no effects on CV_w_ or CV_r_ were observed.

### Dataset 2

Bland-Altman analysis showed fair repeatability of gCBF measurements, but showed also much wider limits of repeatability in long-term compared to intra-session repeated measurements (Fig 2C and 2D). Mean gCBF on day 2 was lower than on day 1 (46.7 vs 54.4, p = 0.045). Analysis of variance components (Table 2) showed that long-term variability is considerably higher than intra-session variability, also when including covariates in the model. CV_t_ decreased by 35% when adjusting for caffeine and hemoglobin. Highly significant inverse associations of gCBF with both hemoglobin (-11.41 [95%CI: -17.63 to -5.20] ml/100ml/min per mmol/l, p = 0.001) and plasma caffeine (-0.25 [95%CI: -0.42 to 0.08] ml/100ml/min per **μ**mol/l, p = 0.006) were found. Excluding measurements with very high caffeine levels (>60 **μ**mol/l), the effects of caffeine remained significant (p = 0.031). Also, a non-significant inverse association of change in gCBF with change in plasma caffeine between the two study days was noted (Fig 3).

## Discussion

Knowledge on the normal variability of cerebral perfusion measurements is crucial when planning and designing studies involving such measurements, and is also important when interpreting studies addressing cerebral perfusion. In order to understand the sources of variability of serial gCBF measurements in more details, we analyzed repeated PCM measurements in healthy volunteers obtained on two separate days. The main findings are that short-term day-to-day variability assessed under strictly controlled conditions in a homogenous population in whom low variability is expected, was found to be small and in the same range as residual intra-session variability. In contrast, long-term variability is much higher when studied in an aged population under less standardized conditions. We also found that from 20% to 35% of the total variability of short-term repeated gCBF measurements can be attributed to important covariates mainly influencing between-subject variability.

The mean and range of both total brain flow and gCBF measurements were similar to those previously reported in studies of young and aged subjects using PCM MRI [7, 18–20]. The mean CBF value in young males (Dataset 1) of 63.4 ml/100ml/min corresponds to 60.4 ml/100g/min when assuming a brain tissue density of 1.05 g/ml [21] which is somewhat higher than the classical text book value of 50 ml/100g/min derived from other techniques [22]. This overestimation is probably related to partial volume effects due to the low resolution of PCM relative to vessel size resulting in overestimation of flow in smaller vessel, in particular in vertebral and basilar arteries [23, 24]. Also, small systematic error may result depending on the level of PCM measurements as flow intended for small extracerebral branches is included when measuring at the level of vertebral arteries (as in Dataset 1), whereas cerebellar braches from the vertebral arteries is not included when measuring at the level of the basilar artery (as in Dataset 2). In the aged population (Dataset 2) we found a mean gCBF of 50.5 ml/100 ml/min (or 48.1 ml/100g/min) which is very similar to the value of 51.2 ml/100ml/min previously reported in a large aged, mixed gender population [20]. The lower gCBF values found in aged subjects compared to younger subjects is thus in agreement with previous publications applying PCM and probably reflects mainly a true age related gCBF decline also reported using a range other techniques [12, 25, 26] and in lesser degree methodological bias.

In terms of intra-session repeatability our results are also in agreement with previous studies [7, 19, 27] confirming excellent intra-session repeatability. As expected, the repeatability between days tended to be poorer compared to sequential same day measurements. Previous studies on PCM day-to-day repeatability have not included duplicate measurements [19, 28], and accordingly could not separate methodological imprecision from true variability. One study of MRI measurements of CMRO_2_ including duplicate PCM measurements performed at five sessions within a two week period reported intra-session and inter-session and inter-subject CV of gCBF to 2.8%, 7.5% and 17.4%, respectively [29]. Due to differences in the calculation of CV, these CV values cannot be compared directly with those reported in the present study.

The present study aimed to quantify true day-to-day variability. In Dataset 1 we found that for short-term repeated measurements this component to be relatively small compared not only to between-subject variability, but also at the same level or slightly lower than the residual intra-session variability. This finding suggests that resting gCBF is relatively constant over time when examined in standardized conditions in a homogenous population with expected low within-subject variability. From Dataset 2 it appears that even when taking important physiological covariates and method imprecision into account, long-term variability estimated in an aged and more heterogeneous population, and applying a less strictly controlled experimental setup, is as expected much higher compared to short-term day-to-day variability. The higher CV_w_ in Dataset 2 may be attributed to a number of factors. Notably, as shown in Fig 2D large gCBF changes >20 ml/100ml/min were observed in three participants suggesting that the higher variability may partially be attributed to large single subject changes related to hormonal [11] or other factors not accounted for in the present analysis. Other potential sources related to the applied techniques, study design and the effects of caffeine are addressed below.

The residual intra-session variability (CV_r_) was as expected lower than between subject variability (CV_b_). The unadjusted gCBF CV_b_ of 9.4% in the Dataset 1 is lower than the values estimated from Dataset 2 including both males and females, whereas it is only slightly lower than the value of 11.6% reported in a recent study also including only young males [24]. Also the CV_r_ of 4% is lower than the values of 7.1% in ref. [7] and of 6.5% reported previously [24]. The lower residual variability is most likely due to the longer acquisition time of the PCM measurements in the present study. Further, it should be noted that in Dataset 2, measurements were performed at the level the basilar artery which may be less straight and less parallel to the ICAs and thus compromise both accuracy and precision.

Variable planning may influence day-to-day variability. In Dataset 1, a screen dump of planning from day 1 was available when planning PCM on day 2, whereas in Dataset 2 planning was based on the general criteria used in our laboratory. A recent study suggested that applying an automatic PCM planning algorithm may reduce residual variability compared to manual planning [28] and could be of particular value in studies involving repeated PCM measurements. It is thus possible that more variable planning may have contributed to the higher CV_w_ in Dataset 2 compared to Dataset 1.

Caffeine withdrawal can increase gCBF and caffeine intake can decrease gCBF [17, 30]; effects that may depends on daily caffeine intake suggesting that cerebral perfusion is adapted to habitual caffeine levels. Therefore, instructing study participants to abstain from their habitual caffeine intake is actually an intervention that might influence the measurements [17, 31]. Both in Dataset 1 and Dataset 2 day-to-day variability (CV_w_) decreased only slightly when including caffeine in the model. As suggested by Fig 3, caffeine abstinence (Dataset 1) appears to effectively reduce plasma caffeine day-to-day variability and associated gCBF changes compared to the spontaneous caffeinated state (Dataset 2), although the association of plasma caffeine change with gCBF change was not significant. It is also remarkable that even if participants were asked to abstain from caffeine before the experiment, plasma caffeine levels up to 10 **μ**mol/l were observed. Whether this reflects lack of compliance or variable metabolism is not clear, but similar observations have been made in patients undergoing myocardial perfusion imaging [32].

Also in agreement with previous studies, we found hemoglobin to an important determinant of cerebral perfusion [14]. With increasing hemoglobin levels, lower gCBF is required to maintain a constant cerebral oxygen delivery (and vice versa). Adjusting for hemoglobin did not reduce residual intra-session variability as hemoglobin was relatively constant. However, in other studies involving interventions that might change hemoglobin or when larger within variability could be expected, e.g. repeated measurements in pre-menopausal women, adjusting for hemoglobin would be relevant.

The present study has some limitations. First of all, sample size in Dataset 1 was relatively small, and in order to minimize the possible gender and age related effects on gCBF [26, 33] we included only young healthy males. For comparison we therefor also included data from a previous study in order to assess the estimates of variability in a dataset where larger variability is expected. Although applying very similar methods in Dataset 1 and Dataset 2, smaller differences related to imaging techniques, participants and in study design prohibit direct pooling of data. Accordingly, it cannot be determined if the larger day-to-day variability in Dataset 2 compared to Dataset 1 is related to the longer interval between study days per se or to other factors such as the population studied or factors related to the study design and the applied techniques.

Secondly, it would have been of interest to include measurement of arterial partial pressure of CO_2_. Spontaneous variations in end-tidal P_CO2_ have been shown to induce in particular intra-subjects gCBF changes in the order of 10–15% per kPa [33, 34]. Data from previous studies from our laboratory [24, 33] shows day-to-day changes in arterial or end-tidal P_CO2_ within ±0.4 kPa corresponding to maximum day-to-day gCBF changes of 4–6%. Assessment of arterial P_CO2_ may obtained by either arterial cannulation or capnography through a closed breathing system, neither of which were considered practical in the experimental setup or generally performed in MRI studies of cerebral perfusion.

An important limitation to the analysis of Dataset 2 is that we derived CV_r_ from another population. It would have been optimal if CV_r_ in Dataset 2 had been determined in the same population using a design similar to that of Dataset 1. As the imaging protocols and setup in ref [7] and in Dataset 2 were very similar, we used the CV_r_ from [7] to estimate CV_w_ in Dataset 2 assuming that CV_r_ would be similar. We cannot know if this assumption holds true, but we believe that the differences in data acquisition in [7] and Dataset 2 are minor and it is unlikely that true CV_r_ in Dataset 2 differs from that in ref [7] to an extend that would alter the main finding of a much larger CV_w_ in Dataset 2 than in Dataset 1.

We believe that the findings of the present study can provide researchers with valuable information for planning futures studies involving gCBF measurements. Using the variance estimates from the Dataset 1, a power calculation shows that in order to detect a 10% difference in gCBF between two groups, a sample size of 21 in each group is required. Performing duplicate measurements and including covariates reduces sample size to 17 and 14, respectively. For within-subject changes, adding duplicate measurements reduces sample size from 8 to 6 in order to detect a 10% change, and adding covariates further reduces the required sample size to 5 subjects. The analyses, however, do also suggest that such low temporal variability may only be achieved when studying a homogenous population under strictly controlled conditions, and that the estimated temporal variability cannot be applied to more heterogeneous populations studied in less well controlled conditions, e.g. large population based studies [2, 20] or studies of aging involving repeated measurements at different time points [2, 35].

In conclusion, our results suggest that the contribution of short-term day-to-day variability to total variability is relatively small compared to between-subject variability of repeated gCBF measurements when performed close in time and under standardized conditions. The study also shows that a substantial fraction of the total variability can be attributed to the effects of easily obtained covariates predominantly by reducing between subject variability. By including covariates and performing duplicate measurements, the ability to detect temporal changes in gCBF may be improved in future studies.

## Supporting information

S1 Table Imaging parameters Dataset 2 and ref. [7]. (DOCX)

S1 Data Dataset 1 and 2. (CSV)
