# Supplementary material for: Components of day-to-day variability of cerebral perfusion measurements – Analysis of phase contrast mapping magnetic resonance imaging measurements in healthy volunteers
Source: PLoS One. 2018 Jun 7;13(6):e0197807. doi: 10.1371/journal.pone.0197807 (PMC5991708; doi:10.1371/journal.pone.0197807)
Supplement: S1 Table — (DOCX) [file pone.0197807.s001.docx]

**S1Table. Imaging parameters Dataset 2 and ref. [7]**

|  |  | Dataset | Ref. [7] |
| --- | --- | --- | --- |
| Head coil | Channels | 32 | 8 |
| 3DT1 | TR /TE | 10 ms / 5 ms | 10 ms / 5 ms |
|  | Flip angle | 8 ° | 8 ° |
|  | Matrix | 240 x 200 | 240 x 200 |
|  | Voxel size | 1x1x1 mm | 1x1x1 mm |
|  | Sensitivity encoding factor | 2 | 2 |
| PCM | TR /TE | 12 ms / 7 ms | 12 ms / 7 ms † |
|  | Flip angle | 10 ° | 10 ° |
|  | Matrix | 320 x 320 | 320 x 320 † |
|  | Voxel size | 0.75x0.75x8 mm | 0.75x0.75x8 mm † |
|  | ECG gating | 20 frames/cycle | 20 frames/cycle |
|  | velocity encoding | 100 | 75 and 150 |

† in four measurements a matrix of 240 x240, TR =9 ms, TE = 6 ms, flip angle 10°, voxel size 1 x1 x 8 mm were used
